# Reduced thermal variability in cities and its impact on honey bee thermal tolerance

**DOI:** 10.7717/peerj.7060

**Published:** 2019-06-07

**Authors:** Karina Sánchez-Echeverría, Ignacio Castellanos, Luis Mendoza-Cuenca, Iriana Zuria, Gerardo Sánchez-Rojas

**Affiliations:** 1 Centro de Investigaciones Biológicas, Universidad Autónoma del Estado de Hidalgo, Mineral de la Reforma, Hidalgo, Mexico; 2 Facultad de Biología, Universidad Michoacana de San Nicolás de Hidalgo, Morelia, Michoacán, Mexico; 3 Laboratorio Nacional de Análisis y Síntesis Ecológica (LANASE-UNAM), Universidad Nacional Autónoma de México, Morelia, Michoacán, Mexico

**Keywords:** Honeybee, *Apis mellifera*, Urbanization, Thermal tolerance, Urban heat island, Physiology, Microclimate, Pollinator, Plasticity, Acclimation

## Abstract

Urbanization is one of the most significant land cover transformations, and while climate alteration is one of its most cited ecological consequences we have very limited knowledge on its effect on species’ thermal responses. We investigated whether changes in environmental thermal variability caused by urbanization influence thermal tolerance in honey bees (*Apis mellifera*) in a semi-arid city in central Mexico. Ambient environmental temperature and honey bee thermal tolerance were compared in urban and rural sites. Ambient temperature variability decreased with urbanization due to significantly higher nighttime temperatures in urban compared to rural sites and not from differences in maximum daily temperatures. Honey bee thermal tolerance breadth [critical thermal maxima (CT_max_)—critical thermal minima (CT_min_)] was narrower for urban bees as a result of differences in cold tolerance, with urban individuals having significantly higher CT_min_ than rural individuals, and CT_max_ not differing among urban and rural individuals. Honey bee body size was not correlated to thermal tolerance, and body size did not differ between urban and rural individuals. We found that honey bees’ cold tolerance is modified through acclimation. Our results show that differences in thermal variability along small spatial scales such as urban-rural gradients can influence species’ thermal tolerance breadths.

## Introduction

Urbanization is one of the most profound drivers of current environmental change (Shochat et al., 2006; Grimm et al., 2008). Currently, more than half of the human population lives in cities, and this figure is expected to rise in the upcoming decades, particularly in developing countries of Africa, Asia and Latin America, resulting in an expansion of urbanized areas worldwide (United Nations, 2014). Thus, it is important to investigate species’ responses to such rapid changes in order to understand the present and future impacts of urbanization. The number of studies targeting the ecological impact of urban areas on biodiversity has increased in the past few years, most of them examining the effect of urbanization on species assemblages with a focus on the role of the urbanized landscape as a filter for the community (Chown & Duffy, 2015; Diamond et al., 2015; Harrison & Winfree, 2015; Ouyang et al., 2018). However, many species can occur in both rural and urban environments, and the consequences of the urban environment on the responses of these populations (e.g., behavioral and physiological) are poorly understood (Diamond et al., 2017; Banaszak-Cibicka et al., 2018).

One of the most distinct consequences of urbanization, and a clear example of anthropogenic microclimate modification, involves a decline in thermal variability and an increase in average ambient temperature in cities when compared to surrounding rural areas (Oke, 1982; Pickett et al., 2001; Gaston, Davies & Edmondson, 2010). This urban heat island effect results primarily from the removal of vegetation and a higher absorption of solar radiation due to impervious surfaces such as buildings, parking lots and roads, and also from direct heat generation related to human activity as well as reduced wind speeds because buildings restrict air circulation (Rizwan, Dennis & Liu, 2008). The duration and magnitude of the temperature differential between urban and rural areas depend on city size, population density and the spatial heterogeneity of the urban landscape, mainly related to the percentage of human-made surfaces (Pickett et al., 2001), and vegetation type and cover (Imhoff et al., 2010). Although urban heat islands have been noticed since 1833 (Howard, 1833), studies of their effects on animal thermal responses are remarkably limited (Brans et al., 2017; Diamond et al., 2017; Hamblin, Youngsteadt & Frank, 2018).

Insects are highly dependent on ambient temperature to ensure their biological functions (Angilletta, 2009). Their capacity for thermal regulation is limited, and their persistence in the environment and resistance to unfavorable temperatures depend in large degree to their thermal tolerance (Chown & Nicolson, 2004). Thermal tolerance is a trait delineated by the coldest and hottest temperatures at which organisms can maintain muscle control (critical thermal minimum, ‘CT_min_’ and critical thermal maximum, ‘CT_max_’, respectively). It can be modified through short-term processes such as phenotypic plasticity (e.g., acclimation) and long-term processes such as evolutionary adaptation (Chown & Nicolson, 2004; Angilletta, 2009). The thermal tolerance breadths of organisms (i.e., the difference between CT_max_ and CT_min_) often match the temperature extremes they are likely to encounter. The evidence supporting this pattern comes from studying species across latitudinal and altitudinal geographical gradients, with thermal tolerance limits being narrower for organisms living in lower latitudes and elevations due to their reduced seasonal and diurnal temperature fluctuations compared to environments with more variable climatic conditions (higher latitudes and elevations) (Janzen, 1967; Chown & Terblanche, 2006; Liefting, Hoffmann & Ellers, 2009; Overgaard et al., 2011; Sunday, Bates & Dulvy, 2011; Araújo et al., 2013).

Honey bees are heterothermic insects that are present across urban and rural environments throughout the world (Cane, 2005) and are being affected by climate change (Le Conte & Navajas, 2008). Individuals are born as strict stenothermic larvae completely dependent on the adult bees for heat (Heinrich, 1993). Newly born adult females perform hive duties in the brood nest where temperatures are maintained near 34 °C (Jones et al., 2005). Honey bee females develop the capacity to generate endothermic heat from muscle contractions and begin foraging after a few days of hatching as adults (Free & Spencer-Booth, 1960; Heinrich, 1993). Foragers are capable of regulating their body temperature across a range of ambient temperatures (Heinrich, 1980). Honey bee foragers lose neuromuscular activity (i.e., enter into chill-coma) when thorax temperatures are below their CT_min_ (∼10 °C) (Esch, 1988; Goller & Esch, 1990a; Goller & Esch, 1990b) and above their CT_max_ (∼50 °C) (Kovac et al., 2014). Thermal breadth can however be altered by plasticity (e.g., acclimation) (Free & Spencer-Booth, 1960; Free & Spencer-Booth, 1962) and evolutionary change (Chuda-Mickiewicz et al., 2009; Kovac et al., 2014) on different time scales. Below their CT_min_, foragers cannot generate heat for colony thermoregulation and flight initiation, and have reduced survival if exposed to these low temperatures for prolonged periods (Free & Spencer-Booth, 1960; Esch, 1988; Goller & Esch, 1990a; Heinrich, 1993). Foragers have lower chill-coma temperature than nurses, drones or queens because they stay away from the brood nest and acclimate to the cooler ambient temperatures in the internal peripheral areas of the hive, particularly at night (Free & Spencer-Booth, 1960; Goller & Esch, 1990a; Goller & Esch, 1991).

We investigated whether reduced thermal variability and an increase in ambient temperature caused by urbanization can influence the limits of thermal tolerance in the honey bee (*Apis mellifera* L.), one of the world’s most important crop pollinators (Klein et al., 2007). We hypothesized that ambient temperature in our urban study site would be less variable and on average higher compared to rural settings. As a consequence honey bees from urban areas would exhibit narrower climatic tolerance breadths, with higher tolerance to extreme warm temperatures and lower tolerance to extreme cold temperatures than bees from rural areas. We also investigated whether the difference in thermal sensitivity between urban and rural bees resulted from an acclimation response. Since larger body size individuals are known to be able to tolerate more extreme temperatures (Pereboom & Beismeijer, 2003; Oyen, Giri & Dillon, 2016; Peters et al., 2016), honey bee body size was measured.

## Materials & Methods

### Site selection

**Figure 1:**
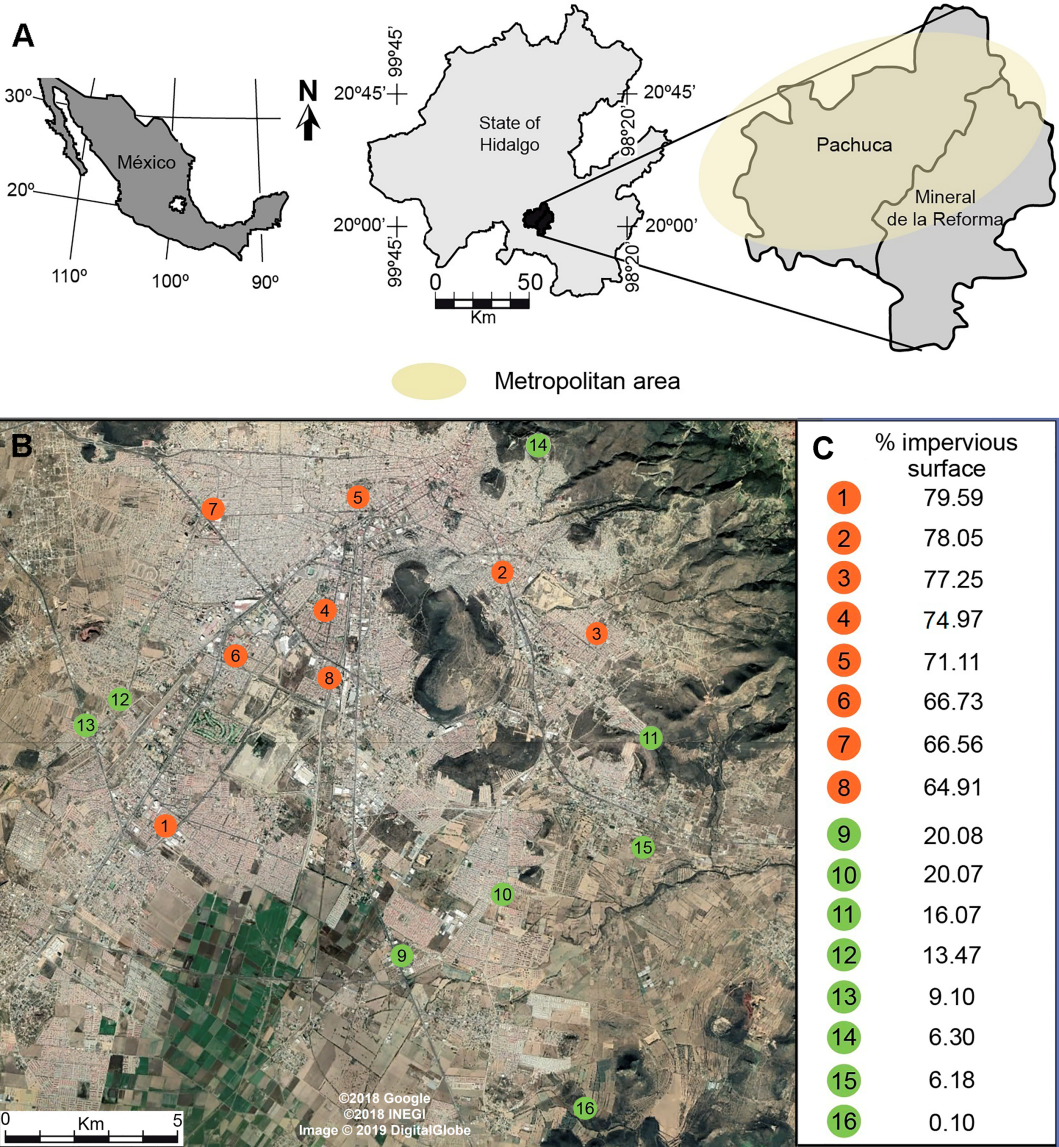
(A) Location of the metropolitan area of Pachuca in the state of Hidalgo, Mexico and (B) the sites where honey bees were collected. (C) The percentage cover of impervious surface at each site is presented on the right for urban (orange) and rural (green) sites; percentages were calculated in 500 m radius buffers centered at each collecting site. The map in Fig. 1A was created using ArcGis (Vers. 10.2, ESRI) and Adobe Illustrator CS5. The satellite image in Fig. 1B was taken from a Google Earth Pro image (©2018 Google, ©2018 INEGI, Image ©2019 Digital Globe).

Our study was conducted in the metropolitan area of Pachuca, Hidalgo, Mexico, which covers approximately 100 km^2^. It is located between 19°50 and 20°10N, and between 98°41 and 98°57W, at 2,400–2,800 m above sea level. It has a population of 512,196 inhabitants, and has one of the fastest growing rates in the country (INEGI, 2010). It has a semi-arid climate, with a mean annual temperature of 15 °C and a mean annual precipitation of 367.6 mm (Gómez-Aíza & Zuria, 2010). We randomly selected 8 urban and 8 rural sites separated by at least 1 km (Fig. 1) (Waddington et al., 1994; Baum et al., 2011; Leonard et al., 2018). We measured the percentage cover of impervious surfaces (houses, buildings, roads, parking places) in urban and rural sites in buffers of 500 m radius, centered at each sampling site, using a 2015 WorldView-2 (Digital Globe) high-resolution satellite image and ArcGis (ver. 10.2, ESRI). For the purpose of this study, we defined urban sites as those having more than 60% impervious cover (72.4 ± 2.1, mean ± se), while rural sites had less than 20% impervious surface (11.4 ± 2.5, mean ± se) (Fig. 1).

### Ambient temperature evaluation

Diurnal temperature variation in urban and rural sites was recorded during the study period because it is a better predictor of thermal tolerance than is annual temperature variation (Sheldon & Tewksbury, 2014; Sheldon, Leaché & Cruz, 2015). Ambient temperature was recorded using 16 temperature dataloggers (Thermochron iButton, model DS1921G). One waterproofed coated datalogger (Roznik & Alford, 2012) was placed in each of the 8 urban and 8 rural selected sites where bees were collected (see below). Dataloggers were placed in full shade, 10 cm above the ground, programmed to record temperatures every 15 min during the bee collecting period. Diurnal temperature variation in urban and rural sites was calculated by subtracting mean minimum temperature from mean maximum temperature.

### Field collection

We sampled feral honey bee foragers (*A. mellifera*) in the 8 urban and 8 rural sites during June 2015, which corresponds to the peak bee foraging period and many of the plant species visited by honey bees are flowering in both urban and rural sites during this period. Also, ambient temperatures resulting from urban heat islands are more pronounced during the summer months (Imhoff et al., 2010). Three randomly selected sites were visited each day under sunny conditions to collect bees from each site between 10:00–14:00, which corresponds to the bee activity period with the lowest temperature variation. We minimized environmental and procedural effects by collecting two bees from each site each day it was visited until reaching at least six individuals (6–12) to test for CT_min_ and at least six individuals (6–12) to test for CT_max_. Honey bees were collected with 250 ml plastic containers and transported to the laboratory at 15 °C to reduce their movement. After being captured, individuals were kept with sucrose (Sigma-Aldrich, S0389) and distilled water (50/50) until the beginning of the experiments (see below). Honey bee collection and treatment followed regulations in Mexico with approval granted from SEMARNAT (License No. FAUT-0174).

### Maximum and minimum temperature limits

We held honey bees in the laboratory at a temperature of 25 °C for at least one hour prior to testing thermal limits to ensure body temperatures were the same before experimental tests. Prior to performing tests, we weighed each honey bee after removing their pollen loads. Honey bee fresh weight was obtained with an electronic balance (Scientech, model ZSA 80) and pollen was removed using a fine detail hair brush. To determine honey bee critical thermal limits, we randomly selected individuals from each of the urban and rural sites to test for either critical thermal maximum (CT_max_) or critical thermal minimum (CT_min_). Seventy four individuals from the urban and 80 from the rural sites were used to test for CT_min_ while 52 bees from the urban and 55 from the rural sites to test for CT_max_. CT_max_ and CT_min_ were measured by placing individual bees inside 25-ml clear plastic cups with a small piece of water-saturated cotton submerged in a temperature-controlled water bath (Julabo, model F25). The water bath temperature was established at 25 °C for both CT_max_ and CT_min_ assays and was increased or decreased at a constant rate of 0.5 °C min^−1^ (Hazell et al., 2008; Hamblin et al., 2017). A thermocouple (Physitemp, model IT-24P) attached to the bee’s thorax was connected to a thermometer (Physitemp, model BAT-12) to register the temperature at which bees lost mobility when the temperature was either raised (CT_max_) (Lutterschmidt & Hutchison, 1997; Hazell et al., 2008) or lowered (CT_min_) (Hazell et al., 2008).

### Acclimation effects on thermal tolerance

In order to determine if honey bees’ thermal limits respond to acclimation, we evaluated if lower thermal tolerances changed under controlled laboratory temperatures. We did not evaluate plasticity in upper thermal limits because previous studies with terrestrial insects have found that narrower thermal tolerance breadths result from changes in CT_min_, rather than in CT_max_ (Addo-Bediako, Chown & Gaston, 2000; Overgaard et al., 2011; Hoffmann, Chown & Clusella-Trullas, 2013; Bozinovic et al., 2014). Bees used in this experiment were obtained from a colony present in one of the urban sites. Capped brood was removed from the urban nest and placed inside an incubator (Shel Lab, model LI15) with 70% relative humidity and 34 °C, and newly emerged adult females were marked and kept under this conditions for a period of three days with sucrose (Sigma-Aldrich, S0389) and distilled water (50/50). Single individuals were then placed inside Petri dishes with sucrose and distilled water (50/50) and were randomly positioned in temperature controlled incubators at either 20 °C (*n* = 10), 24 °C (*n* = 12), or 34 °C (*n* = 12) with 70% relative humidity for a period of four days. A period of four days was chosen because honey bees need at least two days to manifest changes in cold tolerance (Free & Spencer-Booth, 1960). We then tested if their tolerance to lower temperatures differed when acclimated to the different temperatures by measuring the time to recover activity after exposure to low temperature (chill-coma recovery time: CCRT). We determined the time required for bees to recover from exposure to 0 °C (Angilletta et al., 2007). Single individuals from different acclimation treatments were placed in a temperature-controlled water bath with a starting temperature of 25 °C and cooled at a constant rate of 0.5 °C min^−1^ until reaching 0 °C; individuals were then observed to determine the time it took them to right themselves at a temperature of 25 °C after having been at 0 °C for 1 min. We also determined the CCRT of a randomly selected control group of three day old adult females (*n* = 12) that had emerged inside an incubator with 70% relative humidity and 34 °C with sucrose and distilled water (50/50).

### Statistical analysis

Urban and rural ambient temperature ranges, maxima, minima, and averages were compared with a *t*-test. To determine whether honey bees from urban sites exhibit narrower temperature tolerance breadths than bees from rural sites, mean CT_min_ was subtracted from mean CT_max_ at each site. Honey bee CT_max_ and CT_min_ for each site was obtained from the averages of 6–12 individuals collected at each site. Thermal tolerance breadths, CT_max_, and CT_min_ from bees collected in urban and rural sites were compared with a *t*-test. Chill-coma recovery times of bees exposed to the different experimental temperatures were compared using a one-way ANOVA and Tukey post-hoc tests were used to compare means (*p* < 0.05). Data was log_10_ transformed when the assumptions of normality and variance homogeneity were not met. To determine if body size was related with honey bee thermal tolerance, we analyzed the relationship between honey bee body weight and CT_min_, CT_max_, and thermal tolerance breadth using a linear regression analysis. We used SigmaStat Vers. 3.5 to perform the statistical analyses.

## Results

### Ambient temperature evaluation

**Figure 2:**
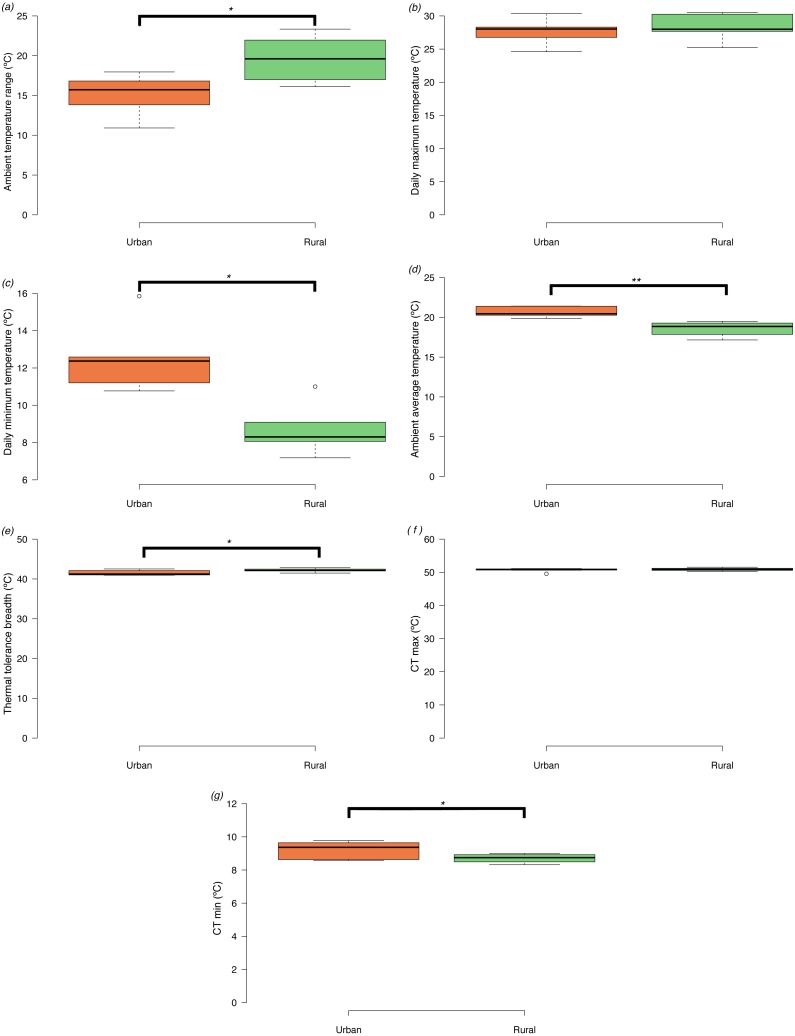
Box plots of ambient temperatures and thermal tolerances of *Apis mellifera* in urban and rural sites (minimum, lower quartile, median, upper quartile and maximum). Tukey-style whiskers were used (Krzywinski & Altman, 2014; Spitzer et al., 2014). (A) Daily ambient temperature ranges (T_max_−T_min_), (B) daily maximum temperatures (T_max_), (C) daily minimum temperatures (T_min_), (D) daily average temperatures (E) thermal tolerance breadths (CT_max_−CT_min_), (F) critical thermal maxima (CT_max_), and (G) critical thermal minima (CT_min_). Asterisks denote significant differences among treatments: *p < 0.05, **p < 0.01.

We could only recover dataloggers or ambient temperature data form 10 of the 16 sites (5 urban and 5 rural). Ambient temperature range size was significantly narrower in urban compared to rural sites (t = −2.45, d.f. = 8, *p* = 0.040) (Fig. 2A). The narrower ambient temperature range in the urban landscape did not result from differences in mean daily maximum temperatures among urban and rural sites (t = −0.54, d.f. = 8, *p* = 0.610) (Fig. 2B), but from differences in higher mean daily minimum temperatures in the urban areas compared to the rural sites (*t* = 3.48, d.f. = 8, *p* = 0.008) (Fig. 2C). Average daily temperatures were significantly higher in urban compared to rural sites (*t* = 3.99, d.f. = 8, *p* = 0.004) (Fig. 2D).

### Maximum and minimum temperature limits and acclimation effects on thermal tolerance

Honey bees from urban sites had significantly narrower tolerance breadths than bees from rural sites (t = −2.53, d.f. = 14, *p* = 0.024) (Fig. 2E). CT_max_ did not differ significantly between urban and rural bees (t = −0.78, d.f. = 14, *p* = 0.448) (Fig. 2F), but CT_min_ (log_10_ transformed) (Fig. 2G) was significantly higher in urban compared to rural bees (*t* = 2.44, d.f. = 14, *p* = 0.029).

**Figure 3:**
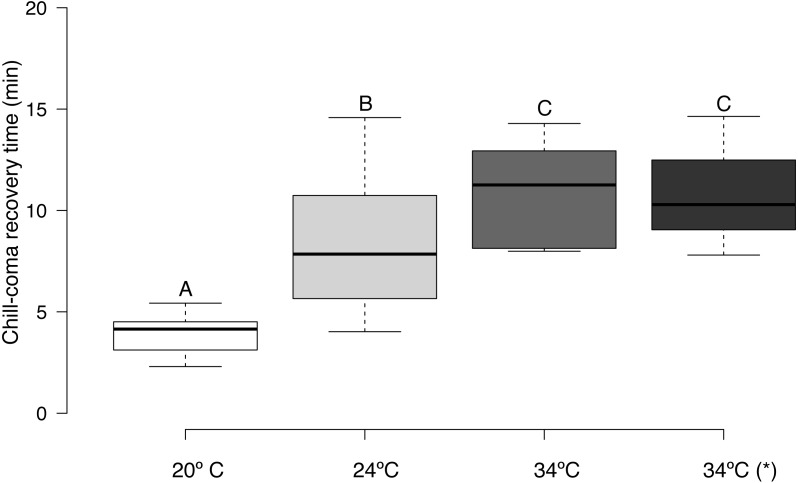
Box plots of chill-coma recovery times of adult *Apis mellifera* subjected to experimental temperatures. Tukey-style whiskers were used (Krzywinski & Altman, 2014; Spitzer et al., 2014). *, thermal tolerance of control group (see text for details). Different letters denote significant differences among treatments (*p* < 0.05).

CCRT (log_10_ transformed) was affected by acclimation temperature (*F* = 30.63, d.f. = 3,45, *p* = 0.0001). It took bees exposed to 20 °C significantly less time to recover from chill-coma than bees exposed to 24 °C, and bees exposed to 20 and 24 °C had significantly lower CCRTs than bees exposed to 34 °C (Fig. 3). There was no significant relationship between honey bee body mass and CT_min_ (*r* = 0.029, *n* = 16, *p* = 0.915), CT_max_ (*r* = 0.023, *n* = 16, *p* = 0.934), and thermal tolerance breadth (*r* = 0.005, *n* = 16, *p* = 0.986). Body weight did not differ significantly between urban and rural bees (*t* = 0.153, d.f. = 8, *p* = 0.881).

## Discussion

The relationship between the temperature extremes to which individuals are exposed and their thermal tolerances has been mainly documented in studies conducted across large-scale temperature gradients, with thermal tolerance breadth being narrower in environments with reduced temperature fluctuations (e.g., lower latitudes and elevations) compared to environments with more variable climatic conditions such as higher latitudes and elevations (Angilletta, 2009; Araújo et al., 2013). Our overall results show that differences in thermal variability along smaller spatial scales such as urban-rural gradients can influence species’ thermal tolerance breadths.

The decrease in ambient temperature variability and increase in average ambient temperature in urban sites compared to rural sites found for the metropolitan area of Pachuca coincide with results documented in other cities (Oke, 1982; Gaston, Davies & Edmondson, 2010; Imhoff et al., 2010). Similar maximum daytime temperatures in urban and rural habitats have been documented for cities with arid and semi-arid climates due to scarce vegetation and the presence of bare soil exposed to solar radiation in the rural landscapes (Shepherd, 2006; Bounoua et al., 2009; Imhoff et al., 2010), as is the case for the open scrub forest in the rural areas of the metropolitan area of Pachuca (Carbó-Ramírez & Zuria, 2011; Sánchez-Echeverría, Castellanos & Mendoza-Cuenca, 2016). Differences in ambient temperature range across urban and rural sites in our study site paralleled those of honey bee thermal tolerance breadth, and resemble a pattern found across large geographic areas (Chown & Terblanche, 2006; Araújo et al., 2013; Sunday et al., 2014). Further, studies along geographical gradients have shown in terrestrial insects that narrower thermal tolerance breadths in more stable climates (lower latitudes and altitudes) result from changes in CT_min_, rather than in CT_max_ (Addo-Bediako, Chown & Gaston, 2000; Overgaard et al., 2011; Hoffmann, Chown & Clusella-Trullas, 2013; Bozinovic et al., 2014), a macrogeographic pattern that parallels the microgeographic pattern observed among honey bees in the metropolitan area of Pachuca.

The empirical studies that have reported that thermal performance and tolerance of terrestrial animals differ between urban and rural sites have documented higher CT_max_ in urban compared to rural individuals (Angilletta et al., 2007; Diamond et al., 2017; Warren, Bayba & Krupp, 2018), which differs from our results with honey bees. The discrepancy between our results and those reported in these studies is likely due in part to differences in daily maximum temperatures among urban and rural habitats. The three aforementioned studies were conducted in cities surrounded by high biomass forest vegetation, where urban habitats tend to have higher maximum temperatures than rural habitats (Imhoff et al., 2010), which can lead to urban individuals having higher thermal tolerances than rural individuals (Angilletta et al., 2007). In contrast, urban and rural sites in arid and semiarid cities with low biomass vegetation tend to have similar maximum ambient temperatures (Imhoff et al., 2010), which could lead to similar higher thermal tolerances in urban and rural individuals, as was found in our study for honey bees. In addition, the maximum ambient temperatures registered in our urban and rural sites were considerably lower than the CT_max_ reported for honey bees (Atmowidjojo et al., 1997; Kovac et al., 2014); this study), while the maximum ambient temperatures registered in the urban sites in the previous three studies did overlap with the CT_max_ reported for ants, which was their study group (Angilletta et al., 2007). More studies investigating the thermal responses of different taxa across cities with different climates will provide more insight into how organisms respond to urban climates (Hamblin et al., 2017; Youngsteadt et al., 2017).

Few studies have reported critical thermal limits of bees, particularly lower critical limits (Free & Spencer-Booth, 1960; Esch, 1988; Goller & Esch, 1990a; Goller & Esch, 1990b; Chuda-Mickiewicz et al., 2009; Oyen, Giri & Dillon, 2016; Peters et al., 2016; Oyen & Dillon, 2018). CT_min_ is related to body size in some bee species (Heinrich, 1993; Oyen, Giri & Dillon, 2016) however, we did not find a relationship between honey bee body mass and critical thermal limits. Differences in nutritional state can influence lower critical thermal limits in bees (Free & Spencer-Booth, 1960), but see (Oyen & Dillon, 2018). However, honey bee foragers’ nutritional condition (amount of carbohydrates, lipids and proteins) does not vary with urbanization in the metropolitan area of Pachuca (Hernández-Medina, 2018) and we did not find that honey bees from urban and rural sites differ in body size (an indicator of environmental quality in bees; Banaszak-Cibicka et al., 2018). Moreover, some studies have suggested that habitat quality in the city might be better for bees than rural landscapes, since the former may provide with richer and year-round food resources, higher and more stable temperatures, and less environmental pollution (Banaszak-Cibicka et al., 2018). Thus, it is unlikely that differences in nutritional state among urban and rural honey bees can explain differences in honey bee thermal tolerances in our study.

Honey bee foragers have 1–2 °C lower chill-coma temperatures than nurse bees as a result of their acclimation to the lower temperatures in the cooler internal peripheral areas of the nest, particularly at night (Free & Spencer-Booth, 1960; Heinrich, 1993). Winter bees also have 1–2 °C lower chill-coma temperatures than summer bees (Free & Spencer-Booth, 1960). Although urban and rural sites were relatively close to each other (3–10 km), we did find a ∼1 °C difference between the CT_min_ of urban and rural bees. Thus, it is likely that the lower night ambient temperature found in our rural sites may account for the lower CT_min_ of rural foragers. This is partially supported by the acclimation effect found in honey bees exposed to laboratory experimental temperatures that differed by 4 °C, which was the difference between the lowest mean temperatures registered in rural and urban sites.

The lower CT_min_ of rural foragers could allow them to generate endothermic heat from muscle contractions more readily at lower temperatures, be more active, and provide heat for colony thermoregulation. In contrast, it is possible that urban bees may not need to invest as much energy and will consume less honey as a result of experiencing higher minimum and similar maximum ambient temperatures (Free & Spencer-Booth, 1958; Free & Spencer-Booth, 1960; Heinrich, 1993; Vollet-Neto, Menezes & Imperatrix-Fonseca, 2011). Since bees provided with artificial nest heating and lower thermal variability produce more brood, honey, and pollen as a result of reducing the energetic costs in colony thermoregulation (Free & Spencer-Booth, 1958; Wineman, Lensky & Mahrer, 2003; Erdogan, Dodologlu & Emsen, 2009; Vollet-Neto, Menezes & Imperatrix-Fonseca, 2011), future studies should be conducted to determine if the microclimates that result from urban heat islands can enhance honey bee colony performance.

## Conclusions

Our results show that differences in thermal variability along small spatial scales such as urban-rural gradients can influence species’ thermal tolerance breadths. We also show that thermal tolerance breadth in urban honey bees can be modified in response to acclimation. Further research addressing the effects of increasing temperatures and decreasing climatic variability caused by urban heat islands on physiological, ecological, and evolutionary processes will permit improved predictions of species responses in urban and future climates.

##  Supplemental Information

10.7717/peerj.7060/supp-1 Data S1 Raw data applied for data analyses and preparation of Figs. 2 and 3

## Additional Information and Declarations

### Field Study Permissions

The following information was supplied relating to field study approvals (i.e., approving body and any reference numbers): Honey bee collection and treatment followed regulations in Mexico with approval granted from SEMARNAT (License No. FAUT-0174).

### Data Availability

The following information was supplied regarding data availability: Raw data measurements are available as a Supplemental File.
